# Microbiome Changes in Humans with Parkinson’s Disease after Photobiomodulation Therapy: A Retrospective Study

**DOI:** 10.3390/jpm12010049

**Published:** 2022-01-05

**Authors:** Brian Bicknell, Ann Liebert, Craig S. McLachlan, Hosen Kiat

**Affiliations:** 1 Faculty of Health Sciences, Australian Catholic University, 40 Edward St, Sydney, NSW 2060, Australia; 2 Research Governance Office, Sydney Adventist Hospital, 185 Fox Valley Rd, Sydney, NSW 2076, Australia; ann.liebert@outlook.com; 3 Faculty of Medicine and Health Science, University of Sydney, Sydney, NSW 2006, Australia; 4 Centre for Healthy Futures, Torrens University, Sydney, NSW 2000, Australia; reperfusion@hotmail.com (C.S.M.); hosen.kiat@chi.org.au (H.K.); 5 Faculty of Medicine, Health and Human Sciences, Macquarie University, Balaclava Rd, Ryde, NSW 2129, Australia; 6 Cardiac Health Institute, Melbourne, NSW 2121, Australia

**Keywords:** microbiome, Parkinson’s disease, photobiomodulation

## Abstract

There is a paucity of information on the effect of photobiomodulation therapy on gut microbiome composition. Parkinson’s disease is a progressive neurological disorder with few management options, although the gut microbiome has been suggested as a potential avenue of treatment. We retrospectively analysed the microbiome from human stool samples from a previously published study, which had demonstrated the efficacy of photobiomodulation to treat Parkinson’s patients’ symptoms. Specifically, we have observed changes in the microbiome of Parkinson’s patients after a 12-week treatment regimen with photobiomodulation to the abdomen, neck, head and nose. Noted were positive changes in the Firmicutes to Bacteroidetes (F:B) ratio, which is often interpreted as a proxy for gut health.

## 1. Introduction

Parkinson’s disease (PD) encompasses a broad range of motor, cognitive and behavioural clinical signs and symptoms, which vary from patient to patient and complicate the treatment of individual PD sufferers. The symptoms of PD are managed with levodopa, often combined with an enzyme inhibitor (carbidopa) to ensure maximum delivery of L-dopa to the brain and subsequent conversion to dopamine by the remaining neurons in the substantia nigra. This medication regimen over time may fail to prevent the progression of the disease; hence, other interventions may be required. Here, we present our data on the use of photobiomodulation applied to the gut and other areas to target the gut-brain axis in Parkinson’s disease.

There has been an increasing understanding of the link between the gut microbiome, the enteric nervous system [1] and a number of diseases, such as kidney disease [2], liver disease [3] and cardiovascular disease [4]. Acknowledgement of the importance of the gut–brain axis has increased the recognition of the link between microbiome balance and brain function. It is appreciated that some bacteria that compose the gut microbiome are associated with a range of behavioural dysfunctions and neurodegenerative diseases [5]. This is especially true in Parkinson’s disease [6]. For example, dysbiosis of the gut microbiome can reduce the number of short chain fatty acid (SCFA) producing bacteria, which in turn increases local inflammatory signalling [7]. Reduction in SCFA production [8], reduced gastrointestinal functional and anatomical integrity and a consequent increase in the movement of bacterial metabolites (e.g., lipopolysaccharide) across the gut wall [9,10] are all features of PD, resulting in increased inflammation [11].

Interestingly, local gut inflammation may act as a trigger for the misfolding and aggregation of α-synuclein in the enteric nervous system [12]. Importantly, α-synuclein has been demonstrated to be transported from the gut to the brain in mice [13,14,15]. Vagotomy has been shown to be protective against PD in mice [15], and truncal vagotomy is somewhat protective in humans [16]. Up to 90% of PD sufferers have gastrointestinal disturbances (most commonly constipation) [17], which often begin years before PD is diagnosed [18]. IBD [19] and IBS [20] appear to be risk factors for PD, and in a retrospective study, Lewy bodies have been detected in gastrointestinal nerve fibres up to 20 years before Parkinson’s disease presentation [21].

Photobiomodulation (PBM) therapy is the use of narrow-wavelength bands of non-thermal light (LED or laser) to modulate cellular responses. PBM targets molecules that absorb light (chromophores), especially cytochrome-C-oxidase in the mitochondria, which increases ATP production, releases reactive oxygen species and promotes increased mitochondrial membrane potential, as well downstream cellular signalling, including gene transcription [22,23]. PBM therapy has a been shown to be a non-invasive and safe therapy, free of deleterious side effects. PBM has a multitude of effects in the body due to its action at a mitochondrial and cellular level [24].

We have previously shown that PBM treatment applied to the abdomen of mice can lead to a beneficial change in the microbiome [25]. More recently, we reported that a combination of PBM treatments delivered to the head, nose, neck and abdomen in humans has the potential to attenuate or reverse some of the clinical signs and non-motor symptoms of PD [26], and similar improvements were also demonstrated with remote PBM treatment to the abdomen and neck, without any transcranial treatment [27]. Information related to the human microbiome composition is, however, lacking.

The objective of this study was to compare two faecal microbiome samples (pre- and post-treatment) from a convenience sample of participants in a PD study before and after they had completed a 12-week course of PBM therapy to the abdominal, head, neck and nasal areas.

## 2. Materials and Methods

The microbiome data were assembled from faecal samples collected during a previously described prospective proof-of-concept study [26] that assessed the effect of PBM on the clinical signs and symptoms of Parkinson’s disease. The study was approved by the Griffith University Human Research Ethics Committee (2018/16), registered with the Australian New Zealand Clinical Trials Registry (Universal Trial number U1111-1205-2035). All participants gave written consent prior to taking part in the study, which included the collection of faecal samples.

### 2.1. Participants

Participant characteristics are given in Table 1. Participants were recruited in January 2019. The participants were males (*n* = 5) and females (*n* = 7) aged between 60 and 80 years (mean age = 70.8, st. dev. 7.79, range 55.6–81.2). All had established diagnosis of idiopathic Parkinson’s disease (by their respective neurologists), stage I to III on the modified Hoehn and Yahr scale [28] and a history of stable (unchanged) anti-Parkinson’s disease medications (if taken) for 3 months prior to entry to the study. All participants were interviewed and examined by a neurologist to ensure eligibility for enrolment into the study and signed a written informed consent form. Enrolment inclusion and exclusion criteria were as previously described [26].

Participants were treated with PBM for 12 weeks as previously described [26,27]. Briefly, participants were treated with a four-diode laser device (904 nm, 30 mW) transdermally over nine points of the abdomen in a grid pattern (3.6 joules per point, 32.4 joules total energy) and over the C1/C2 region of the neck (7.6 joules total energy) as well as transcranially with four LED diodes (240 joules total energy) and intranasally with a single LED diode (15 joules total energy). Total treatment time was 30 min. Participants were treated three times per week for 4 weeks, followed by twice per week for 4 weeks and then once per week for 4 weeks (24 total treatments). Full PBM parameters are provided in Supplementary Table S1. The treatment protocol used Class 1 lasers and LEDs, with no need for safety glasses.

### 2.2. Sample Collection

Participants were instructed to not change their dietary habits or day-to-day activities for the duration of the study. Faecal samples were self-collected by study participants before the PBM treatment began (pre-treatment) and after 12 weeks of treatment (post-treatment) was completed. Sample were stored frozen at −20 °C until the extraction of DNA.

### 2.3. Microbiome Analysis

Genomic DNA was extracted and purified using QIAamp PowerFecal Pro DNA Kit (Qiagen-Venlo, The Netherlands) following the manufacturer’s instructions, except that tubes were heated to 90 °C for 5 min before the bead beating step to increase DNA yield. Genomic DNA was quantified using a Qubit^®^ Fluorometer, and approximately 10 ng/μL of the purified DNA sample was sent to the Australian Genomic Research Facility (www.argf.org.au; accessed 26 August 2019) for amplification of the V3–V4 hypervariable region of 16S rRNA gene to target bacteria and archaea using primers 514f (5′-GTGCAGAATTGCCCTATCC-3′) and 806r (5′-GACTACHVGGGTATCTAATCC-3′) and for next-generation sequencing (NGS) using the MiSeq platform (Illumina^®^—San Diego, CA, USA).

Generated sequences were analysed for metagenomic bacterial diversity using the Quantitative Insights into Microbial Ecology 2 (QIIME2) pipeline (version 2021.8; open-source software; www.qiime2.org) [29] following suggestions on the qiime2 website (https://docs.qiime2.org/2019.1/; last accessed 19 March 2021). Demultiplexed paired-end reads fastq sequences were imported using Casava 1.8 paired-end demultiplexed fastq format. Primers and barcodes were removed, sequences were quality trimmed to 280 bp, denoised and chimeras were removed (consensus method) using DADA2. Amplicon sequence variants (ASVs) were aligned with mafft [30] (via q2-alignment) and used to construct a phylogeny with fasttree2, using q2-phylogeny [31]. Taxonomy was assigned to ASVs using the q2-feature-classifier [32] based on Greengenes (version 13_8) at 99% OTUs, trained using a naïve Bayes classifier [33].

Microbiome community structure was analysed using alpha diversity (within sample richness) and beta diversity (between sample similarity), calculated using the q2-diversity plug-in at a rarefaction of 30,000 sequences sampling depth. Alpha diversity was assessed using the Shannon, Simpson, Fisher alpha, Kulsinski, Chao1, Faith_pd and Lladser_pe indices. Beta diversity was assessed using PERMANOVA (with 999 iterations) using weighted and unweighted UniFrac distances. The analysis of composition of microbiomes (ANCOM) was used to identify any taxa driving changes. Individual genera were further examined based on their relative occurrence in samples. Genera were only included if they represented at least 0.5% of the total microbiome in more than 25% of samples. Differences between pre-treatment and post-treatment samples were judged to be substantial if a greater than log2 fold change occurred. Genera were flagged if twice as many participants showed an increase as showed a decrease or if twice as many participants showed a decrease as showed an increase.

## 3. Results

All study participants and caregivers reported no major deviation from their usual diet and activities of daily living during the 12-week study period. A total of 24 faecal samples were included from the 12 participants, before and after 12 weeks of PBM treatment. From these samples, 4,537,843 sequencing reads were obtained. The sequences were denoised to 1,539,775 sequences and grouped into 2939 separate “features” with between 30,193 and 90,956 features per sample.

**Figure 1:**
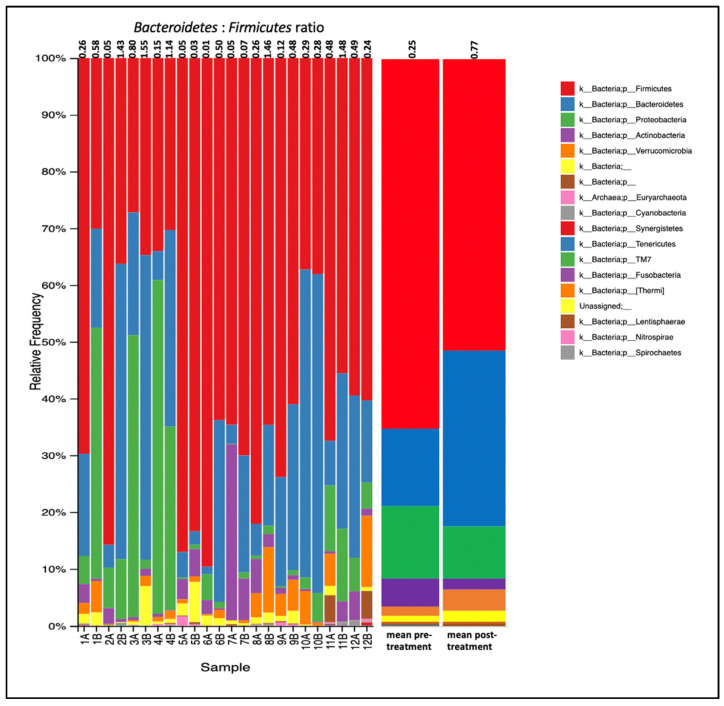
Stacked bar chart indicating the relative abundances of phyla detected in the gut microbiomes of PD participants before (pre-) and after 12 weeks (post-) of PBM treatment. A = pre-treatment; B = post-treatment.

**Figure 2:**
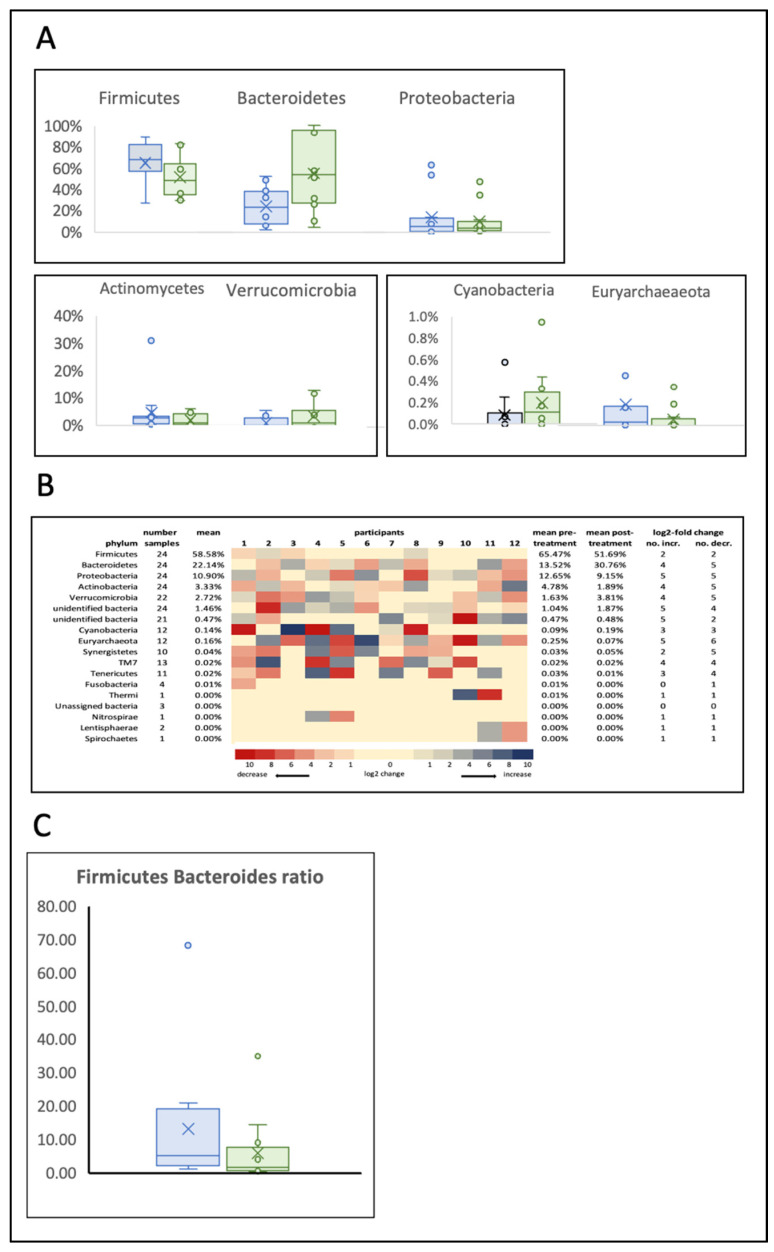
Changes in the abundance of phyla in the gut microbiome of PD participants after 12 weeks of treatment with PBM. (**A**) Abundance of phyla. (**B**) Heatmap of changes in phyla for individual participants. (**C**) Firmicutes:Bacteroidetes ratio.

The most abundant phyla (Figure 1) were Firmicutes (62.64%), Bacteroidetes (22.14%), Proteobacteria (10.99%), Actinobacteria (3.48%) and Verrucomicrobia (3.44%). The remaining phyla combined accounted for less than 2.5% of the microbiota. The relative abundance of Firmicutes, Proteobacteria and Actinobacteria decreased after treatment, and the relative abundances of Bacteroidetes and Verrucomicrobia increased after treatment (Figure 2A). The average Firmicutes:Bacteroidetes ratio was 4.60 before treatment and 1.58 after treatment, with 9 of 12 participants showing a decreased ratio (Figure 2C). Equal numbers of participants showed a log2 fold increase or a log2 fold decrease in abundance in both Firmicutes and Bacteroidetes phyla (Figure 2B).

**Figure 3:**
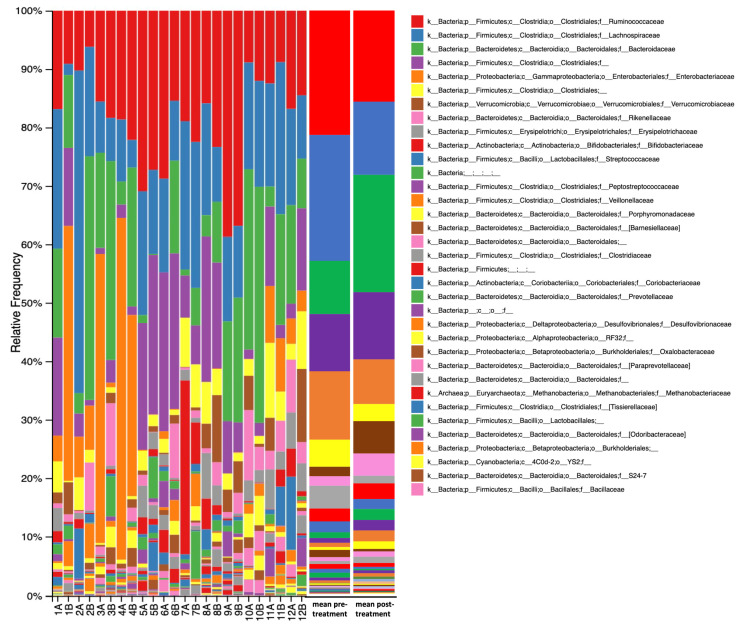
Stacked bar chart indicating the relative abundances of the 35 most abundant genera detected in the gut microbiomes of PD participants before (pre-) and after 12 weeks (post-) of PBM treatment. A = pre-treatment; B = post-treatment.

**Figure 4:**
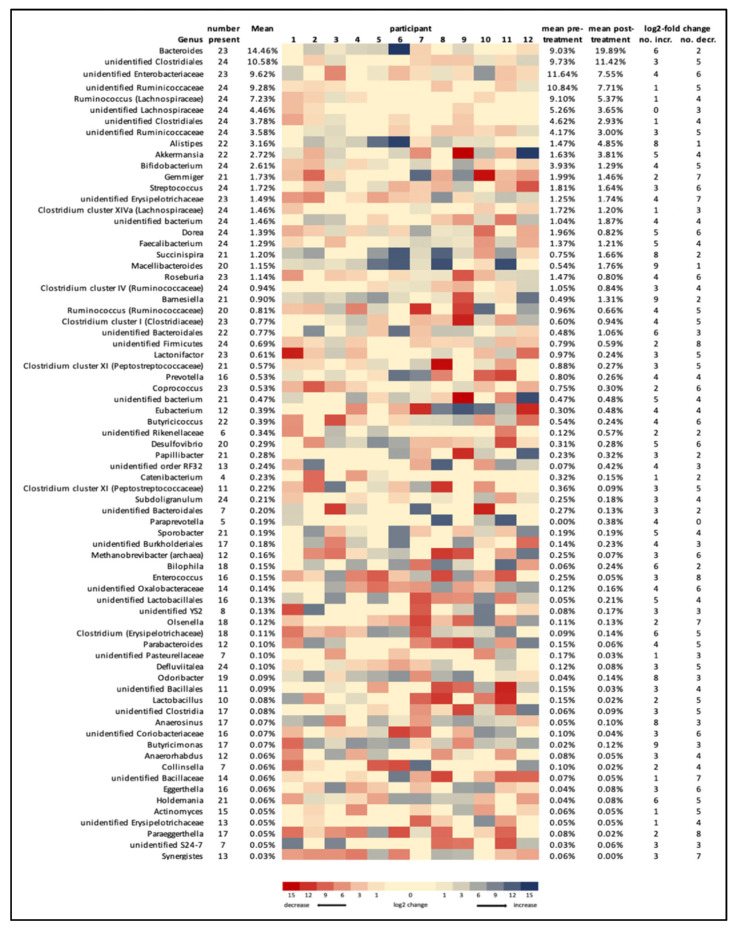
Heatmap of changes in relative abundance of genera for individual participants. Colours represent the change in relative abundance expressed as log2 fold change of post-treatment compared to pre-treatment.

The total number of genera detected was 172 (Figure 3), with the 10 most common genera accounting for over 60% of all genera and the 20 most common genera accounting for 82%. Almost half of the 172 genera detected (76, or 44%) could not be identified to genus level, including 6 of the 10 most common genera. The changes in microbiome composition are shown as a heatmap in Figure 4 for the most abundant genera. For all participants, 59.5% of the genera showed a greater than log2 fold change, 24.9% of the genera showed a greater than 5 log2 fold change and 4.8% showed a greater than 10 log2 fold change, post-treatment compared to pre-treatment. Genera that were flagged as increased (>log2 fold increase in twice as many participants as those that showed a decrease) included a number of Bacteroidetes genera (*Bacteroides*, *Alistipes, Macellibacteroides, Barnesiella, Odoribacter* and an unidentified Bacteroidales genus). Non-Bacteroidetes genera flagged as increased included *Paraprevotella*, *Succinispira, Bilophila, Anaerosinus,* and *Anaerotruncus.* Genera that were flagged as decreased (>log2 fold decrease) included *Gemmiger, Clostridium* cluster XI (2 genera), *Coprococcus, Methanobrevibacter* (archaea), *Enterococcus, Eggerthella, Paraeggerthella, Olsenella, Lactonifactor, Actinomyces* and *Synergistes,* as well as unidentified genera from Ruminococcaceae, Bacillaceae, Coriobacteriaceae, Erysipelotrichaceae and Firmicutes. No phyla, family or genera was statistically different between the pre- and post-treatment as measured by the ANCOM statistic.

**Figure 5:**
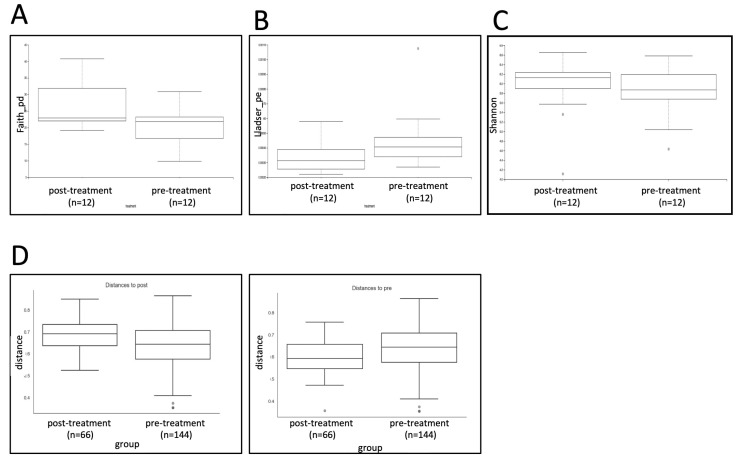
(**A**) Faith’s_pd measure of alpha diversity (*p* < 0.1). (**B**) Lladser_pe measure of alpha diversity (*p* < 0.1). (**C**) Shannon measure of alpha diversity (*p* = NS). (**D**) Unweighted UniFrac measure of beta diversity (*p* = NS).

Alpha diversity as measured using multiple indices, including the Shannon index, did not show a significant change between pre- and post-treatment, with the exception of Faith’s Phylogenic Diversity (Faith’s_pd) index and Lladser point estimate (Lladser_pe) index, which were both significant at *p* < 0.1 (Figure 5). Beta diversity, as measured by the unweighted UniFrac statistic, did not indicate a significant difference between pre- and post-treatment (*p* = NS).

## 4. Discussion

To our knowledge, this is the first study that has demonstrated that the application of PBM is potentially capable of altering the microbiome in individuals with Parkinson’s disease. Our study supports previous work that has shown that PBM produces beneficial changes in the gut microbiome in a mouse model of PD [25] and produces favourable changes in gut microbiome diversity in a patient undergoing radiotherapy and immunotherapy for breast cancer [34], with an increase in the number of known beneficial bacteria and a decrease in the number of potentially pathogenic genera.

The changes seen in the microbiome at the phylum and genus levels could not be attributed to significant changes in any taxa, when tested with ANCOM. There was, however, an array of changes in individual microbial taxa after PBM treatment, with some phyla and numerous families and genera showing either an increase or a decrease of greater than log2 fold in many of the participants.

The changes seen at the phylum level (decreased Firmicutes, increased Bacteroidetes) are reflected as a change in the Firmicutes to Bacteroidetes (F:B) ratio. It is frequently reported that a higher ratio is characteristic of poorer gut health and is associated with obesity and an increased inflammatory state [35] and ageing [36] although not all studies have found this [37]. While an association of this ratio with neurodegeneration is worthy of consideration and the ratio was found to be reduced in patients with amyotrophic lateral sclerosis [38,39] and depression [40], a review paper [41] evaluating microbiomes among PD sufferers found no significant differences in the F:B ratio.

To date, four non-pharmacotherapeutic interventions have been suggested to slow the progression of PD via manipulation of the microbiome [42], these being diet, pro- and prebiotics, antibiotics and faecal microbiota transplant [43]. Based on our findings, PBM is a potential novel fifth intervention and may complement new and existing treatment strategies. PBM using laser light represents a non-invasive, safe alternative to target microbiome changes. PBM has been demonstrated over many years to be safe for a variety of medical conditions, including neurodegenerative diseases and traumatic brain injury [44] as opposed to PD medications, which are frequently associated with a multitude of side effects, adversely affecting quality of life [45]. PBM treatment remote from the site of an injury or disease has also been shown to be effective, potentially by activating stem cells [46,47], circulating cell-free functional mitochondria [48], circulating chemical messengers or “mitokines” [49] and/or through immune modulation [24]. To this list, we might now add changing the microbiome as a potential mechanism. Remote treatment is especially significant in PD, since the site of neuronal damage in the brain (substantia nigra) is beyond the limit that light can readily penetrate when delivered transcranially and the gut microbiome is often suggested as an appropriate target for PD therapy. As a potential therapy, PBM would ideally be commenced as early as possible in the disease trajectory, before the severe reduction or complete elimination of beneficial bacteria from the microbiome (including by medications) and may best be combined with diet, pre- and probiotics or faecal microbiota transplant to restore microbiome genera.

There are numerous cross-sectional studies that report significant differences in the abundance of certain bacteria taxa between PD and the general population, although as yet, there is no universally accepted microbiome signature of PD. Nonetheless, a list of bacterial genera can be compiled that are either underrepresented or overrepresented in the gut microbiome of PD sufferers. Multiple clinical studies have shown higher abundances of *Akkermansia, Bifidobacterium,* Ruminococcaceae and *Lactobacillus* as well as potential pathogens such as *Streptococcus* and genera of the family Enterobacteriaceae, such as *Escherichia–Shigella*, *Enterococcus* and *Proteus* [6,10,50,51,52,53,54]. Other genera that have been shown to be increased in PD sufferers include *Oscillibacter, Porphyromonas*, *Anaerococcus, Megasphaera* and the archeon *Methanobrevibacter* [6,50,53]. Genera that produce SCFAs, which generally are recognised as being beneficial to the gut microbiome, and which have been shown to be reduced among PD patients include *Bacteroides*, *Clostridium* cluster IV, *Faecalibacterium*, *Roseburia*, *Moryella* and genera of the family Leptospiraceae [6,50,53].

Although changes in the microbiome can occur over the timescale of hours, related to diurnal rhythms and food intake, and over days, related to diet change and xenobiotic ingestion (including medications) [55], the microbiome of healthy adults can remain stable over long timescales [56,57,58]. It might be expected, however, that a dysfunctional microbiome associated with PD would worsen as the disease progressed. Notwithstanding the multiple studies that have compared the microbiome of PD and healthy controls, few studies have assessed changes in microbiome in PD over time. One study [59] did not find significant changes in the microbiome of a small cohort (29 participants) over a 2-year period, although there was a suggestion that *Prevotella* was negatively associated with disease progression. An earlier study on 36 PD sufferers [60], using real-time PCR rather than next generation sequencing, found that lower counts of *Bifidobacterium, Bacteroides*, and *Clostridium* cluster VI were associated with a more rapid deterioration. A 3-year follow up of 25 PD sufferers found an association between the lower levels of *Roseburia* and *Faecalibacterium* and higher levels of Actinobacteria and *Oscillospira* with worsening motor, non-motor and cognitive symptoms [61].

In our study, we have shown that although PBM treatment over 12 weeks did not result in significant changes to the microbiome composition, there was a trend towards a reduction in certain genera and an increase in others. These changes were highly individual, which is unsurprising given the individuality of even healthy microbiomes [62], as well as the individual nature of PD symptoms, individual medication regimens, the stage of disease and participant response to the PBM therapy and reduction of symptoms.

Some of the trends seen in our present study are associated with an increase in beneficial bacteria. One general trend is the increase in genera within the order Bacteroidiales. The genera *Bacteroides*, *Alistipes, Barnesiella*, *Macellibacteroides, Odoribacter* and an unidentified Bacteroidales genera were all flagged as having an increase in many participants. Bacteroidales are considered to be anti-inflammatory, are producers of SCFAs and are more common in the microbiomes of people with high-fibre diets [63]. The genus *Bacteroides* is generally considered a component of a healthy microbiome, being increased in high-fibre diets and decreased in high-fat diets [64] and is generally found to be decreased in the microbiomes of PD sufferers [52,65,66,67]. A positive association between SCFAs and *Bacteroides* has been observed in microbiomes of control groups but not in PD patients [59]. *Barnesiella* and *Alistipes* are also considered to be components of a healthy microbiome [68] and a reduction in *Odoribacter* has been associated with various metabolic diseases [69]. Other (non-Bacterodiales) genera that showed a trend to increase included the SCFA-producing genera *Paraprevotella,* which can be reduced in PD patients [54,70], and *Succinispira.*

The Ruminococcaceae and Lachnospiraceae families have genera that produce SCFAs and are considered anti-inflammatory and beneficial to the human microbiome. These include the *Ruminococcus, Roseburia, Faecalibacterium, Butyricicoccus* and *Gemmiger* genera as well as *Clostridium* cluster IV and *Clostridium* cluster XIVa [71]. In the PD microbiome, these groups have been found to be increased in some studies (especially the Lachnospiraceae) and decreased in others [6]. In our study, these genera were generally increased and decreased in equal proportion, except that *Gemmiger* showed a trend towards decreasing in participants. Although *Gemmiger* is a butyrate producer and is often found in the microbiome along with *Roseburia*, it has also been associated with some cancers, Crohn’s disease relapse and non-alcoholic fatty liver disease [72].

*Lactobacillus* and *Bifidobacterium* are usually recognised as probiotic genera and have been shown to be anti-inflammatory in the gut and to be beneficial in a variety of conditions, including stress, anxiety, autism and depression [73]. Somewhat paradoxically, both have often been reported as being increased in PD [6], including in a recent study that analysed large PD microbiome datasets [54] and a recent meta-analysis [53]. In our study, *Lactobacillus* was present at low levels in the gut microbiomes of our PD participants (0.08% on average) and was decreased in participants after PBM treatment. Approximately equal numbers of participants showed an increase as showed a decrease of *Bifidobacterium* after PBM treatment.

Many bacterial genera that have been shown in multiple (cross-sectional) studies to be increased in PD microbiomes and might be considered as potential signatures of PD [6,67] were either not found in our study (e.g., *Oscillibacter, Megasphaera, Porphyromonas*) or were found in very low numbers (<0.01%) such as *Cloacibacillus*, *Anaerotruncus* and *Anaerococcus*. The archaeon *Methanobrevibacter*, which is generally recognised to increase in PD [53] and also shown to increase at each Hoehn and Yahr stage [51], showed a trend to decrease in our participants.

Several studies have advanced the proposition that an increase in pathogenic or potentially pathogenic bacteria can be characteristic of the PD gut microbiome [54,74,75] and genera acknowledged as detrimental have been found in some studies to be increased in PD. *Clostridium* cluster XI is a group of potential pathogens, which includes *Clostridium difficile*, that is associated with a high-fat diet and type 2 diabetes [76]. In our study, two genera were identified within this group, and both genera showed a trend to decrease in participants following PBM treatment. This trend was also observed for other potential pathogens: *Streptococcus, Enterococcus, Actinomyces, Eggerthella* and the closely related *Paraeggerthella.*

Given the importance of altered gut microbiota in PD sufferers, any improvement in the bacterial balance has the potential to assist in the stabilisation of PD symptoms. The observed changes to the gut microbiome of participants in this study, whose clinical signs and symptoms also improved with PBM, lend support to the important role of microbiome changes in PD. PBM has been shown to alter the microbiome in a mouse model, both in healthy mice [25] and in animal models of Alzheimer’s disease [77,78] and osteoporosis [79]. In humans, one possibility is that the change in the microbiome with PBM could be a primary effect, either acting directly on the bacteria or as a result of the anti-inflammatory effect of PBM, counteracting inflammation in the gut [80] and, hence, reducing dysbiosis-induced gut leakage.

A second possibility is that the changed microbiome is a secondary effect of improvement in the symptoms of PD and the subsequent communication from the brain to the gut. It is interesting to note in this context that there is a suggestion that deep brain stimulation can change the gut microbiome [81]. The end result of either possibility is a changed, possibly healthier, microbiome, which would have positive effects for the trajectory of the disease. The mode of action of PBM to change the microbiome merits further investigation.

This study was preliminary in nature and suffers from a number of limitations, including the lack of a control group, small numbers and heterogeneity of the participants with regard to Parkinson’s symptoms, as well as a lack of information on dietary habits of the participants. These shortcomings would be addressed in a future larger-scale study. Despite these limitations, our results suggest that PBM treatment can influence the microbiome in Parkinson’s disease.

## 5. Conclusions

We have seen changes in the microbiome of Parkinson’s patients after a 12-week treatment regimen with PBM. Specifically, the F:B ratio, which is often interpreted as a proxy for gut health, improved for the majority of participants with PBM treatment. While there were no significant changes in microbial diversity and microbial taxa after PBM treatment, there was, however, a trend toward microbiome changes, including increases in some SCFA-producing bacteria, increases in genera recognised as beneficial to the microbiome and decreases in potential pathogens and some bacteria recognised as harmful to the microbiome. The microbiome of people with PD is complex, highly individual and potentially influenced by many factors such as diet, lifestyle and medications, as well as disease state, comorbidities and stage of Parkinson’s disease. Investigation of the response of the microbiome to PBM treatment is worthy of further study in prospective, controlled clinical trials, in order to confirm the relationship of PBM and microbiome changes in PD patients and investigate the potential of targeting the gut microbiome with PBM as an avenue into the treatment of PD.

## Figures and Tables

**Table 1:** Demographic data of participants in the PBM trial.

Participant	Sex	Age	Hoehn and Yahr Stage	Height	Weight	MDS UPDRS Score	Dominant Hand	Affected Side	Sample Collection	
									Pre- Treatment	Post- Treatment
1	M	71	2	178	78.7	89	R	L	6 January 2019	31 March 2019
2	F	74	2	165	68.3	31	R	L	6 January 2019	31 March 2019
3	F	78	3	156	76.0	57	R	L	6 January 2019	31 March 2019
4	M	75	2	177	75.5	52	R	L	6 January 2019	31 March 2019
5	M	67	2	173	78.6	53	R	L	6 January 2019	31 March 2019
6	M	63	1	175	76.1	36	L	L	6 January 2019	31 March 2019
7	F	53	2	150	48.3	53	R	L	31 March 2019	7 July 2019
8	F	72	2	160	61.7	70	R	L	31 March 2019	7 July 2019
9	F	57	2	169	53.0	42	R	R	31 March 2019	7 July 2019
10	M	69	1	180	77.6	29	R	L	31 March 2019	7 July 2019
11	F	61	2	167	67.7	36	L	R	31 March 2019	7 July 2019
12	F	71	2	163	61.4	67	R	R	31 March 2019	7 July 2019

## Data Availability

The sequence dataset for the gut microbiome of for this study can be accessed at the National Centre for Biotechnology Information (NCBI) Bioproject PRJNA790457: Photobiomodulation Changes to Parkinson’s Microbiome (http://www.ncbi.nlm.nih.gov/bioproject/790457; last accessed 19 December 2021).

## Supplementary Materials

The following supporting information can be downloaded at: https://www.mdpi.com/article/10.3390/jpm12010049/s1. Table S1: Parameters of the photobiomodulation devices and treatment used in the study; Supplementary Data: Contribution of taxa to the microbiome.

## References

[1] Giuffrè M., Moretti R., Campisciano G., Da Silveira A.B.M., Monda V.M., Comar M., Di Bella S., Antonello R.M., Luzzati R., Crocè L.S. (2020). You Talking to Me? Says the Enteric Nervous System (ENS) to the Microbe. How Intestinal Microbes Interact with the ENS. J. Clin. Med..

[2] Wang Y.F., Zheng L.J., Liu Y., Ye Y.B., Luo S., Lu G.M., Gong D., Zhang L.J. (2019). The gut microbiota-inflammation-brain axis in end-stage renal disease: Perspectives from default mode network. Theranostics.

[3] Giuffrè M., Campigotto M., Campisciano G., Comar M., Crocè L.S. (2020). A story of liver and gut microbes: How does the intestinal flora affect liver disease? A review of the literature. Am. J. Physiol. Gastrointest. Liver Physiol..

[4] van den Munckhof I.C., Kurilshikov A., Ter Horst R., Riksen N.P., Joosten L.A.B., Zhernakova A., Fu J., Keating S., Netea M.G., De Graaf J. (2018). Role of gut microbiota in chronic low-grade inflammation as potential driver for atherosclerotic cardiovascular disease: A systematic review of human studies. Obes. Rev..

[5] Cryan J.F., O’Riordan K.J., Cowan C.S., Sandhu K.V., Bastiaanssen T.F., Boehme M., Codagnone M.G., Cussotto S., Fulling C., Golubeva A.V. (2019). The microbiota-gut-brain axis. Physiol. Rev..

[6] Bullich C., Keshavarzian A., Garssen J., Kraneveld A., Perez-Pardo P. (2019). Gut Vibes in Parkinson’s Disease: The Microbiota-Gut-Brain Axis. Mov. Disord. Clin. Pract..

[7] Rizzatti G., Lopetuso L.R., Gibiino G., Binda C., Gasbarrini A. (2017). Proteobacteria: A Common Factor in Human Diseases. BioMed Res. Int..

[8] Unger M.M., Spiegel J., Dillmann K.U., Grundmann D., Philippeit H., Bürmann J., Faßbender K., Schwiertz A., Schäfer K.H. (2016). Short chain fatty acids and gut microbiota differ between patients with Parkinson’s disease and age-matched controls. Park. Relat. Disord..

[9] Forsyth C.B., Shannon K.M., Kordower J.H., Voigt R.M., Shaikh M., Jaglin J.A., Estes J.D., Dodiya H.B., Keshavarzian A. (2011). Increased intestinal permeability correlates with sigmoid mucosa alpha-synuclein staining and endotoxin exposure markers in early Parkinson’s disease. PLoS ONE.

[10] Chiang H.-L., Lin C.-H. (2019). Altered Gut Microbiome and Intestinal Pathology in Parkinson’s Disease. J. Mov. Disord..

[11] Lin C.H., Chen C.C., Chiang H.L., Liou J.M., Chang C.M., Lu T.P., Chuang E.Y., Tai Y.C., Cheng C., Lin H.Y. (2019). Altered gut microbiota and inflammatory cytokine responses in patients with Parkinson’s disease. J. Neuroinflamm..

[12] Caggiu E., Arru G., Hosseini S., Niegowska M., Sechi G., Zarbo I.R., Sechi L.A. (2019). Inflammation, Infectious Triggers, and Parkinson’s Disease. Front. Neurol..

[13] Holmqvist S., Chutna O., Bousset L., Aldrin-Kirk P., Li W., Bjorklund T., Wang Z.-Y., Roybon L., Melki R., Li J.-Y. (2014). Direct evidence of Parkinson pathology spread from the gastrointestinal tract to the brain in rats. Acta Neuropathol..

[14] Kim S., Kwon S.-H., Kam T.-I., Panicker N., Karuppagounder S.S., Lee S., Lee J.H., Kim W.R., Kook M., Foss C.A. (2019). Transneuronal Propagation of Pathologic α-Synuclein from the Gut to the Brain Models Parkinson’s Disease. Neuron.

[15] Uemura N., Yagi H., Uemura M.T., Hatanaka Y., Yamakado H., Takahashi R. (2018). Inoculation of α-synuclein preformed fibrils into the mouse gastrointestinal tract induces Lewy body-like aggregates in the brainstem via the vagus nerve. Mol. Neurodegener..

[16] Liu B., Fang F., Pedersen N.L., Tillander A., Ludvigsson J.F., Ekbom A., Svenningsson P., Chen H., Wirdefeldt K. (2017). Vagotomy and Parkinson disease: A Swedish register–based matched-cohort study. Neurology.

[17] Fasano A., Visanji N.P., Liu L.W., Lang A.E., Pfeiffer R.F. (2015). Gastrointestinal dysfunction in Parkinson’s disease. Lancet Neurol..

[18] Scheperjans F., Derkinderen P., Borghammer P. (2018). The Gut and Parkinson’s Disease: Hype or Hope?. J. Parkinson’s Dis..

[19] Brudek T. (2019). Inflammatory Bowel Diseases and Parkinson’s Disease. J. Park. Dis..

[20] Fu P., Gao M., Yung K.K.L. (2019). Association of Intestinal Disorders with Parkinson’s Disease and Alzheimer’s Disease: A Systematic Review and Meta-Analysis. ACS Chem. Neurosci..

[21] Stokholm M.G., Danielsen E.H., Hamilton-Dutoit S., Borghammer P. (2016). Pathological α-synuclein in gastrointestinal tissues from prodromal Parkinson disease patients. Ann. Neurol..

[22] Hamblin M.R. (2018). Mechanisms and Mitochondrial Redox Signaling in Photobiomodulation. Photochem. Photobiol..

[23] Benson P., Kim J.Y., Riveros C., Camp A., Johnstone D.M. (2020). Elucidating the time course of the transcriptomic response to photobiomodulation through gene co-expression analysis. J. Photochem. Photobiol. B Biol..

[24] Hamblin M.R. (2017). Mechanisms and applications of the anti-inflammatory effects of photobiomodulation. AIMS Biophys..

[25] Bicknell B., Liebert A., Johnstone D., Kiat H. (2018). Photobiomodulation of the microbiome: Implications for metabolic and inflammatory diseases. Lasers Med. Sci..

[26] Liebert A., Bicknell B., Laakso E.-L., Heller G., Jalilitabaei P., Tilley S., Mitrofanis J., Kiat H. (2021). Improvements in clinical signs of Parkinson’s disease using photobiomodulation: A prospective proof-of-concept study. BMC Neurol..

[27] Liebert A., Bicknell B., Laakso E.-L., Jalilitabaei P., Tilley S., Kiat H., Mitrofanis J. (2021). Remote Photobiomodulation Treatment for the Clinical Signs of Parkinson’s Disease: A Case Series Conducted During COVID-19. Photobiomodul. Photomed. Laser Surg..

[28] Bhidayasiri R., Tarsy D. (2012). Parkinson’s disease: Hoehn and Yahr scale. Movement Disorders: A Video Atlas.

[29] Bolyen E., Rideout J.R., Dillon M.R., Bokulich N.A., Abnet C.C., Al-Ghalith G.A., Alexander H., Alm E.J., Arumugam M., Asnicar F. (2019). Reproducible, interactive, scalable and extensible microbiome data science using QIIME 2. Nat. Biotechnol..

[30] Katoh K., Standley D.M. (2013). MAFFT multiple sequence alignment software version 7: Improvements in performance and usability. Mol. Biol. Evol..

[31] Price M.N., Dehal P.S., Arkin A.P. (2010). FastTree 2—Approximately Maximum-Likelihood Trees for Large Alignments. PLoS ONE.

[32] Bokulich N.A., Dillon M.R., Zhang Y., Rideout J.R., Bolyen E., Li H., Albert P.S., Caporaso J.G. (2018). q2-longitudinal: Longitudinal and Paired-Sample Analyses of Microbiome Data. mSystems.

[33] McDonald D., Price M.N., Goodrich J., Nawrocki E.P., DeSantis T.Z., Probst A., Andersen G.L., Knight R., Hugenholtz P. (2012). An improved Greengenes taxonomy with explicit ranks for ecological and evolutionary analyses of bacteria and archaea. ISME J..

[34] Bicknell B., Laakso E.-L., Liebert A., Kiat H. (2022). Modifying the microbiome as a potential mechanism of photobiomodulation: A case report. Photobiomodul. Photomed. Laser Surg..

[35] Stojanov S., Berlec A., Štrukelj B. (2020). The influence of probiotics on the Firmicutes/Bacteroidetes ratio in the treatment of obesity and inflammatory bowel disease. Microorganisms.

[36] Vaiserman A., Romanenko M., Piven L., Moseiko V., Lushchak O., Kryzhanovska N., Guryanov V., Koliada A. (2020). Differences in the gut Firmicutes to Bacteroidetes ratio across age groups in healthy Ukrainian population. BMC Microbiol..

[37] Magne F., Gotteland M., Gauthier L., Zazueta A., Pesoa S., Navarrete P., Balamurugan R. (2020). The Firmicutes/Bacteroidetes Ratio: A Relevant Marker of Gut Dysbiosis in Obese Patients?. Nutrients.

[38] Fang X., Wang X., Yang S., Meng F., Wang X., Wei H., Chen T. (2016). Evaluation of the Microbial Diversity in Amyotrophic Lateral Sclerosis Using High-Throughput Sequencing. Front. Microbiol..

[39] Zhai C.D., Zheng J.J., An B.C., Huang H.F., Tan Z.C. (2019). Intestinal microbiota composition in patients with amyotrophic lateral sclerosis: Establishment of bacterial and archaeal communities analyses. Chin. Med. J..

[40] Saji N., Niida S., Murotani K., Hisada T., Tsuduki T., Sugimoto T., Kimura A., Toba K., Sakurai T. (2019). Analysis of the relationship between the gut microbiome and dementia: A cross-sectional study conducted in Japan. Sci. Rep..

[41] Gerhardt S., Mohajeri M.H. (2018). Changes of Colonic Bacterial Composition in Parkinson’s Disease and Other Neurodegenerative Diseases. Nutrients.

[42] Brown E.G., Goldman S.M. (2020). Modulation of the Microbiome in Parkinson’s Disease: Diet, Drug, Stool Transplant, and Beyond. Neurotherapeutics.

[43] Borody T., Bienenstock J. (2019). The Role of Fecal Microbiota Transplantation in Neurological Diseases. The Microbiome and the Brain.

[44] Cassano P., Caldieraro M.A., Norton R., Mischoulon D., Trinh N.-H., Nyer M., Dording C., Hamblin M.R., Campbell B., Iosifescu D.V. (2019). Reported Side Effects, Weight and Blood Pressure, After Repeated Sessions of Transcranial Photobiomodulation. Photobiomodul. Photomed. Laser Surg..

[45] Jankovic J., Tan E.K. (2020). Parkinson’s disease: Etiopathogenesis and treatment. J. Neurol. Neurosurg. Psychiatry.

[46] Ganeshan V., Skladnev N.V., Kim J.Y., Mitrofanis J., Stone J., Johnstone D.M. (2019). Pre-conditioning with Remote Photobiomodulation Modulates the Brain Transcriptome and Protects Against MPTP Insult in Mice. Neuroscience.

[47] Blatt A., Elbaz-Greener G.A., Tuby H., Maltz L., Siman-Tov Y., Ben-Aharon G., Copel L., Eisenberg I., Efrati S., Jonas M. (2016). Low-Level Laser Therapy to the Bone Marrow Reduces Scarring and Improves Heart Function Post-Acute Myocardial Infarction in the Pig. Photomed. Laser Surg..

[48] Al Amir Dache Z., Otandault A., Tanos R., Pastor B., Meddeb R., Sanchez C., Arena G., Lasorsa L., Bennett A., Grange T. (2020). Blood contains circulating cell-free respiratory competent mitochondria. FASEB J..

[49] Kim B., Brandli A., Mitrofanis J., Stone J., Purushothuman S., Johnstone D.M. (2017). Remote tissue conditioning—An emerging approach for inducing body-wide protection against diseases of ageing. Ageing Res. Rev..

[50] Lubomski M., Tan A.H., Lim S.-Y., Holmes A.J., Davis R.L., Sue C.M. (2019). Parkinson’s disease and the gastrointestinal microbiome. J. Neurol..

[51] Baldini F., Hertel J., Sandt E., Thinnes C.C., Neuberger-Castillo L., Pavelka L., Betsou F., Krüger R., Thiele I., Aguayo G. (2020). Parkinson’s disease-associated alterations of the gut microbiome predict disease-relevant changes in metabolic functions. BMC Biol..

[52] Li F., Wang P., Chen Z., Sui X., Xie X., Zhang J. (2019). Alteration of the fecal microbiota in North-Eastern Han Chinese population with sporadic Parkinson’s disease. Neurosci. Lett..

[53] Romano S., Savva G.M., Bedarf J.R., Charles I.G., Hildebrand F., Narbad A. (2021). Meta-analysis of the Parkinson’s disease gut microbiome suggests alterations linked to intestinal inflammation. npj Park. Dis..

[54] Wallen Z.D., Appah M., Dean M.N., Sesler C.L., Factor S.A., Molho E., Zabetian C.P., Standaert D.G., Payami H. (2020). Characterizing dysbiosis of gut microbiome in PD: Evidence for overabundance of opportunistic pathogens. npj Park. Dis..

[55] Uhr G.T., Dohnalová L., Thaiss C.A. (2019). The Dimension of Time in Host-Microbiome Interactions. mSystems.

[56] Faith J.J., Guruge J.L., Charbonneau M., Subramanian S., Seedorf H., Goodman A.L., Clemente J.C., Knight R., Heath A.C., Leibel R.L. (2013). The Long-Term Stability of the Human Gut Microbiota. Science.

[57] Fu B.C., Randolph T.W., Lim U., Monroe K.R., Cheng I., Wilkens L.R., Le Marchand L., Lampe J.W., Hullar M.A. (2018). Temporal Variability and Stability of the Fecal Microbiome: The Multiethnic Cohort Study. Cancer Epidemiol. Biomark. Prev..

[58] Sunagawa S., Schloissnig S., Arumugam M., Forslund K., Mitreva M., Tap J., Zhu A., Waller A., Mende D.R., Kultima J.R. (2014). Individuality and temporal stability of the human gut microbiome. Cent. Asian J. Glob. Health.

[59] Aho V.T., Houser M.C., Pereira P.A., Chang J., Rudi K., Paulin L., Hertzberg V., Auvinen P., Tansey M.G. (2021). Relationships of gut microbiota, short-chain fatty acids, inflammation, and the gut barrier in Parkinson’s disease. Mol. Neurodegener..

[60] Minato T., Maeda T., Fujisawa Y., Tsuji H., Nomoto K., Ohno K., Hirayama M. (2017). Progression of Parkinson’s disease is associated with gut dysbiosis: Two-year follow-up study. PLoS ONE.

[61] Cilia R., Piatti M., Cereda E., Bolliri C., Caronni S., Ferri V., Cassani E., Bonvegna S., Ferrarese C., Zecchinelli A.L. (2021). Does Gut Microbiota Influence the Course of Parkinson’s Disease? A 3-Year Prospective Exploratory Study in de novo Patients. J. Park. Dis..

[62] Falony G., Joossens M., Vieira-Silva S., Wang J., Darzi Y., Faust K., Kurilshikov A., Bonder M.J., Valles-Colomer M., Vandeputte D. (2016). Population-level analysis of gut microbiome variation. Science.

[63] Fernández J., Redondo-Blanco S., Gutiérrez-Del-Río I., Miguélez E.M., Villar C.J., Lombó F. (2016). Colon microbiota fermentation of dietary prebiotics towards short-chain fatty acids and their roles as anti-inflammatory and antitumour agents: A review. J. Funct. Foods.

[64] Senghor B., Sokhna C., Ruimy R., Lagier J.-C. (2018). Gut microbiota diversity according to dietary habits and geographical provenance. Hum. Microbiome J..

[65] Hegelmaier T., Lebbing M., Duscha A., Tomaske L., Tönges L., Holm J.B., Nielsen H.B., Gatermann S.G., Przuntek H., Haghikia A. (2020). Interventional Influence of the Intestinal Microbiome Through Dietary Intervention and Bowel Cleansing Might Improve Motor Symptoms in Parkinson’s Disease. Cells.

[66] Hasegawa S., Goto S., Tsuji H., Okuno T., Asahara T., Nomoto K., Shibata A., Fujisawa Y., Minato T., Okamoto A. (2015). Intestinal Dysbiosis and Lowered Serum Lipopolysaccharide-Binding Protein in Parkinson’s Disease. PLoS ONE.

[67] Petrov V.A., Saltykova I.V., Zhukova I.A., Alifirova V.M., Zhukova N.G., Dorofeeva Y.B., Tyakht A.V., Kovarsky B.A., Alekseev D.G., Kostryukova E.S. (2017). Analysis of Gut Microbiota in Patients with Parkinson’s Disease. Bull. Exp. Biol. Med..

[68] Kulagina E., Efimov B.A., Maximov P.Y., Kafarskaia L.I., Chaplin A., Shkoporov A. (2012). Species Composition ofBacteroidalesOrder Bacteria in the Feces of Healthy People of Various Ages. Biosci. Biotechnol. Biochem..

[69] Hiippala K., Barreto G., Burrello C., Diaz-Basabe A., Suutarinen M., Kainulainen V., Bowers J.R., Lemmer D., Engelthaler D.M., Eklund K.K. (2020). Novel *Odoribacter splanchnicus* Strain and Its Outer Membrane Vesicles Exert Immunoregulatory Effects in vitro. Front. Microbiol..

[70] Scheperjans F., Aho V., Pereira P.A., Koskinen K., Paulin L., Pekkonen E., Haapaniemi E., Kaakkola S., Eerola-Rautio J., Pohja M. (2015). Gut microbiota are related to Parkinson’s disease and clinical phenotype. Mov. Disord..

[71] Guo P., Zhang K., Ma X., He P. (2020). *Clostridium* species as probiotics: Potentials and challenges. J. Anim. Sci. Biotechnol..

[72] Lang S., Martin A., Farowski F., Wisplinghoff H., Vehreschild M.J., Liu J., Krawczyk M., Nowag A., Kretzschmar A., Herweg J. (2020). High Protein Intake Is Associated With Histological Disease Activity in Patients With NAFLD. Hepatol. Commun..

[73] Wang H., Lee I.-S., Braun C., Enck P. (2016). Effect of Probiotics on Central Nervous System Functions in Animals and Humans: A Systematic Review. J. Neurogastroenterol. Motil..

[74] Karunaratne T.B., Okereke C., Seamon M., Purohit S., Wakade C., Sharma A. (2020). Niacin and Butyrate: Nutraceuticals Targeting Dysbiosis and Intestinal Permeability in Parkinson’s Disease. Nutrients.

[75] Rani L., Mondal A.C. (2021). Unravelling the role of gut microbiota in Parkinson’s disease progression: Pathogenic and therapeutic implications. Neurosci. Res..

[76] Ohashi Y., Fujisawa T. (2019). Analysis of *Clostridium* cluster XI bacteria in human feces. Biosci. Microbiota Food Health.

[77] Wang M., Cao J., Gong C., Amakye K., Yao M., Ren J. (2021). Exploring the microbiota-Alzheimer’s disease linkage using short-term antibiotic treatment followed by fecal microbiota transplantation. Brain Behav. Immun..

[78] Chen Q., Wu J., Dong X., Yin H., Shi X., Su S., Che B., Li Y., Yang J. (2021). Gut flora-targeted photobiomodulation therapy improves senile dementia in an Aß-induced Alzheimer’s disease animal model. J. Photochem. Photobiol. B Biol..

[79] Lu Y., Yang J., Dong C., Fu Y., Liu H. (2021). Gut microbiome-mediated changes in bone metabolism upon infrared light exposure in rats. J. Photochem. Photobiol. B Biol..

[80] Yoshimura T.M., Sabino C.P., Ribeiro M. (2016). Photobiomodulation reduces abdominal adipose tissue inflammatory infiltrate of diet-induced obese and hyperglycemic mice. J. Biophotonics.

[81] Lubomski M., Xu X., Holmes A.J., Yang J.Y.H., Sue C.M., Davis R.L. (2021). The impact of device-assisted therapies on the gut microbiome in Parkinson’s disease. J. Neurol..

