# Supplementary material for: Microbiome Changes in Humans with Parkinson’s Disease after Photobiomodulation Therapy: A Retrospective Study
Source: J Pers Med. 2022 Jan 5;12(1):49. doi: 10.3390/jpm12010049 (PMC8778696; doi:10.3390/jpm12010049)
Supplement: Supplementary file 1 [file jpm-12-00049-s001.zip › jpm-1510531-Supplementary Table S1.pdf]

Table S1. Parameters of the photobiomodulation devices and treatment regimens used in the study.

PARAMETER	IRRADIA 904 nm LASER	VIELIGHT NEURO GAMMA		
Manufacturer	Spectra Analytic Irradia AB	Vielight Inc.		
Diodes	904nm laser diodes (GaAs)	5 x LED diodes		
Wavelength	904nm	810 nm		
Laser class	1	-		
		posterior	anterior	nasal
Number of diodes	4	3	1	1
Output power	30 mW	100 mW	75 mW	25mW
Peak power	25,000 mW			
Pulse frequency	50 Hz	40 Hz	40 Hz	40 Hz
Beam spot size	0.635 cm^2	~1 cm	~1 cm	~1 cm
Power density per diode	47 mW/cm^2	100 mW/cm^2	75 mW/cm^2	25 mW/cm^2
Total output power	120 mW	400 mW		
Irradiation time per point	30 s	2100 s		
Total irradiation time	330 s	2100 s		
Total energy per point	3.6 J	60 J	45 J	15 J
Number of sites	11 (9 abdomen, 2 neck)	3	1	1
Total energy dose per treatment	39.6 J	180 J	45 J	15 J
Treatment frequency				
weeks 1 to 4	3 x per week for 4 weeks	3 x per week for 4 weeks		
weeks 5 to 8	2 x per week for 4 weeks	2 x per week for 4 weeks		
weeks 9 to 12	1 x per week for 4 weeks	1 x per week for 4 weeks		
